# Supplementary material for: Interventions across the Retirement Transition for Improving Well-Being: A Scoping Review
Source: Int J Environ Res Public Health. 2020 Jun 17;17(12):4341. doi: 10.3390/ijerph17124341 (PMC7344699; doi:10.3390/ijerph17124341)
Supplement: Supplementary file 1 [file ijerph-17-04341-s001.zip › Online supplement 1 Search strategies.docx]

**Online supplement 1: Search strategies**

1."retirement"[MeSH Terms] OR "retirement"[All Fields]

2. "Early Medical Intervention"[Mesh]

3. program[All Fields]

4. "health planning"[MeSH Terms]

5. "policy"[MeSH Terms]

6. "intervention"[All Fields]

7. 2-6 OR

8. "health"[MeSH Terms] OR "health"[All Fields]

9. "health status"[MeSH Terms] OR health status[Text Word]

10. wellbeing[All Fields] OR "well being"[All Fields]

11. "quality of life"[MeSH Terms] OR "quality of life"[All Fields]

12. 8-11 OR

13. systematic[sb]

14. Clinical Trial[ptyp] OR Randomized Controlled Trial[ptyp] OR trial[tiab]

15. Comparative Study[ptyp] OR Observational Study[ptyp]

16. "cohort studies"[MeSH Terms] OR ("cohort"[tiab]

17. 13-16 OR

18. 1 AND 7 AND 12 AND 17
