# Supplementary material for: Interventions across the Retirement Transition for Improving Well-Being: A Scoping Review
Source: Int J Environ Res Public Health. 2020 Jun 17;17(12):4341. doi: 10.3390/ijerph17124341 (PMC7344699; doi:10.3390/ijerph17124341)
Supplement: Supplementary file 1 [file ijerph-17-04341-s001.zip › Online supplement 2 MMAT assessment of experimental designs.rtf]

Online supplement 2: MMAT assessment of experimental designs.
RANDOMIZED CONTROLLED TRIALS	
Author, year	Are there clear research questions?	Do the collected data allow to address the research questions?	Is randomization appropriately performed?	Are the groups comparable at baseline?	Are there complete outcome data?	Are outcome assessors blinded to the intervention provided?	Did the participants adhere to the assigned intervention?	
Ashe, 2015	YES	YES	YES	Can't tell	YES	Can't tell	YES	
Van Dyck, 2016	YES	YES	YES	YES	YES	NO	NO	
Stancliffe, 2015	YES	YES	NO	YES	YES	NO	YES	
Fries, 1993	YES	YES	YES	YES	YES	Can't tell	YES	
Fries, 1994	YES	YES	YES	YES	YES	Can't tell	YES	
Lapierre, 2007	YES	YES	NO	Can't tell	YES	Can't tell	YES	
Lara, 2016	YES	YES	YES	Can't tell	YES	YES	YES	
Cunningham, 1987	YES	YES	Can't tell	YES	YES	NO	YES	
Werkman, 2010	YES	YES	YES	YES	YES	Can't tell	YES	
NON-RANDOMIZED STUDIES	
Author, year	Are there clear research questions?	Do the collected data allow to address the research questions?	Are the participants representative of the target population?	Are measurements appropriate regarding both the outcome and intervention (or exposure)?	Are there complete outcome data?	Are the confounders accounted for in the design and analysis?	During the study period, is the intervention administered (or exposure occurred) as intended?	
Dubé, 2005	YES	YES	YES	YES	Can't tell	Can't tell	YES	


 MMAT: Mix Methods Appraisal Tool
